# Telemedicine in medical education: An example of a digital preparatory course for the clinical traineeship – a pre-post comparison

**DOI:** 10.3205/zma001567

**Published:** 2022-09-15

**Authors:** Lina Vogt, Michelle Schmidt, Andreas Follmann, Andrea Lenes, Martin Klasen, Saša Sopka

**Affiliations:** 1 RWTH Aachen, Medizinische Fakultät, AIXTRA - Kompetenzzentrum für Training und Patientensicherheit, Aachen, Germany; 2 RWTH Aachen, Medizinische Fakultät, Uniklinik RWTH Aachen, Klinik für Anästhesiologie, Aachen, Germany

**Keywords:** telemedicine, clinical traineeship preparation course, digitalization of education, medical education

## Abstract

**Introduction: **Telemedicine is a significant component of healthcare in most disciplines, giving great importance to the education of young physicians in this field. However, the topic of telemedicine has not yet been implemented in medical schools' curricula.

This paper makes an important contribution to closing this gap by designing, implementing and evaluating a course with telemedical components. Using the example of a clinical traineeship preparation course, we investigated the extent to which integrated telemedical modules can contribute to the subjective confidence of students with regard to knowledge and confidence in performing practical telemedical skills, such as doctor-patient communication, taking medical histories, and applying handover techniques.

**Project description: **The course evaluation was descriptive. Subjective confidence in clinical telemedicine skills was assessed before and after completion of the course using an online questionnaire and calculated in a pre-post design using Wilcoxon's signed-rank test.

**Results: **The course was rated “very good” (31%) and “good” (54.2%) by the vast majority of students. The results of the Wilcoxon test show significant increases in students' feelings of confidence in performing practical telemedicine skills for all items.

**Discussion: **This study shows that telemedicine modules integrated in a digital preparatory course contribute positively to students' subjective confidence in terms of knowledge and confidence in performing practical telemedicine skills. Specifically, this paper illustrates that professional digital doctor-patient communication, digital documentation of a medical history, and handoff techniques can be learned through telemedicine course content.

**Conclusion: **Telemedicine modules increase students' subjective confidence in performing practical telemedicine skills. Practical telemedicine course content can thus reduce uncertainty in the use of telemedicine and prepare future physicians for its use.

## Zusammenfassung

**Einleitung: **Telemedizin ist in den meisten Fachdisziplinen ein wichtiger Baustein des Gesundheitswesens. Dementsprechend bedeutsam ist die Ausbildung junger Ärzte und Ärztinnen in diesem Bereich. Dennoch ist das Thema Telemedizin in den Curricula medizinischer Fakultäten bisher wenig bis gar nicht implementiert.

Diese Arbeit leistet durch die Konzeption, Durchführung und Evaluation eines Kurses mit telemedizinischen Bestandteilen einen wichtigen Beitrag zum Schluss dieser Lücke. Untersucht wurde am Beispiel eines Famulaturvorbereitungskurses, inwieweit integrierte telemedizinische Module zur subjektiven Sicherheit der Studierenden hinsichtlich Wissen und Sicherheit bei der Ausübung praktischer telemedizinischer Fertigkeiten wie der Arzt-Patienten Kommunikation, Anamneseerhebung und Übergabetechniken beitragen können.

**Projektbeschreibung: **Die Evaluation des Kurses erfolgte deskriptiv. Es wurde die subjektive Sicherheit hinsichtlich der Ausübung klinischer telemedizinischer Fertigkeiten vor Beginn und nach Absolvieren des Kurses mittels Online-Fragebogen erhoben und in einem Prä-Post-Design mittels Vorzeichen-Rang Test (signed-rank test) nach Wilcoxon berechnet.

**Ergebnisse: **Der Kurs wurde von der überwiegenden Mehrzahl der Studierenden mit den Schulnoten „sehr gut“ (31%) und „gut“ (54,2%) bewertet. Die Ergebnisse des Wilcoxon- Tests zeigen für alle Items signifikante Anstiege des Sicherheitsgefühls der Studierenden bei der Ausübung praktischer telemedizinischer Fertigkeiten.

**Diskussion: **Die vorliegende Arbeit zeigt, dass in einem digitalen FamV integrierte telemedizinische Module zur subjektiven Sicherheit der Studierenden hinsichtlich des Wissens und Sicherheit bei der Ausübung praktischer telemedizinischer Fertigkeiten positiv beitragen. Insbesondere verdeutlicht diese Arbeit, dass eine professionelle digitale Arzt-Patienten-Kommunikation, digitale Erhebung einer Anamnese und Übergabetechniken durch telemedizinische Kursinhalte erlernt werden können.

**Schlussfolgerung: **Telemedizinische Module erhöhen die subjektive Sicherheit der Studierenden bei der Ausübung praktischer telemedizinischer Fertigkeiten. Praktische telemedizinische Kursinhalte können somit Unsicherheiten im Umgang mit Telemedizin abbauen und zukünftige Ärzte und Ärztinnen auf ihren Einsatz vorbereiten.

## 1. Introduction

Today, telemedicine is an important component of the healthcare system [1], [2]. Various uses facilitate the interdisciplinary exchange of information, networking of interprofessional groups, and direct contact between patients and between physicians. Telemedicine is now represented in virtually all specialties and ranges from video-based doctor's appointments through teleconsultations to telemedicine as part of emergency medical services [3], [4]. For instance, telemedical emergency physicians have been a permanent fixture in emergency medical services since 2014 and are currently deployed for routine missions in many German regions [5], [6], [7].

As a consequence, the education of young physicians in this area is important to prepare them for the specific changes that result from the inclusion of telemedicine in routine medical practice. The integration of telemedical content early in medical education is being called for not only by the Deutsche Gesellschaft für Telemedizin (German Society for Telemedicine), but also in the National Catalogue of Competency-based Learning Objectives in Undergraduate Medical Education (NKLM 2.0 VII.2-13.1.5), [8], [https://www.nklm.de]. Despite this, the topic of telemedicine in medical education in Germany has not yet been sufficiently implemented and is hardly present in medical schools’ curricula.

This paper makes an important contribution to closing this gap by designing, implementing and evaluating a course with telemedical components. The content of the course was based on a preparatory course for the clinical traineeship. This course has been offered by the medical school at RWTH Aachen University since the 2005 summer semester [9] to give fourth-semester medical students practical training in the relevant healthcare tasks prior to their first clinical traineeship. To teach aspects of telemedicine in a targeted fashion, content with telemedical application was selected from the existing course and adapted to a telemedical setting and for digital implementation. This encompassed, for example, digital communication between patient and physician during video-based appointments, online case history taking, and digital handover.

The present study investigates the extent to which integrated telemedical modules can contribute to the subjective confidence of students with regard to knowledge and confidence in performing practical telemedical skills, such as doctor-patient communication, taking medical histories, and handover techniques.

### Hypothesis

A digital clinical traineeship preparation course with telemedical modules increases the subjective confidence of students with regard to performing practical telemedical skills. 

## 2. Project description

### 2.1. Course design

Before the course started, all of the required materials were made available to the attendees on the e-learning platform Moodle [https://moodle.rwth-aachen.de/]. These materials included all of the learning materials for the anamnesis and handover training, as well as the example cases (cases A1-5) and checklists ISBAR, SAMPLER, MIST and ABCDE (see attachment 1 ). Patient handovers contain risks, such as information loss, which can endanger patient safety. Structured handovers using checklists counteract this and thus increase patient safety [10], [11].

The course began by imparting theoretical principles in a lecture on communication, error management and patient safety. Following this, telemedical content regarding anamnesis and medical handover was taught. The attendees also learned about the different standardized checklists and handover tools.

The students were then divided into eight small groups (breakout rooms) with a maximum of six participants. At this point, the attendees presented their paper-based cases (A1-5) to the group (see attachment 2 ). Each of the eight small groups was connected to an instructor who had been given instructions in advance (see attachment 3 and attachment 4 ).

Practical application of the previously learned material took place in the next step. Each small group was connected with a simulated patient (SP), for whom a standardized case history on a specific medical issue was taken, with the learning objective of structuring a professional consultation with the patient. The SP had been closely trained in the SP program and received the case details and instructions in advance. There were two patient cases in total (see attachment 5 ), for which four groups worked on case A and four groups on case B. Under supervision in each of the small groups, the students practiced establishing a doctor-patient relationship online and taking a structured, patient-centered case history. The groups were reconfigured after all of the participants had completed their case history taking with the SP. The attendees were divided into a total of four larger groups in which case A and case B were represented so that the next step involved handovers of the cases by the participants. The groups were each guided by two medical educators.

**Figure 1:**
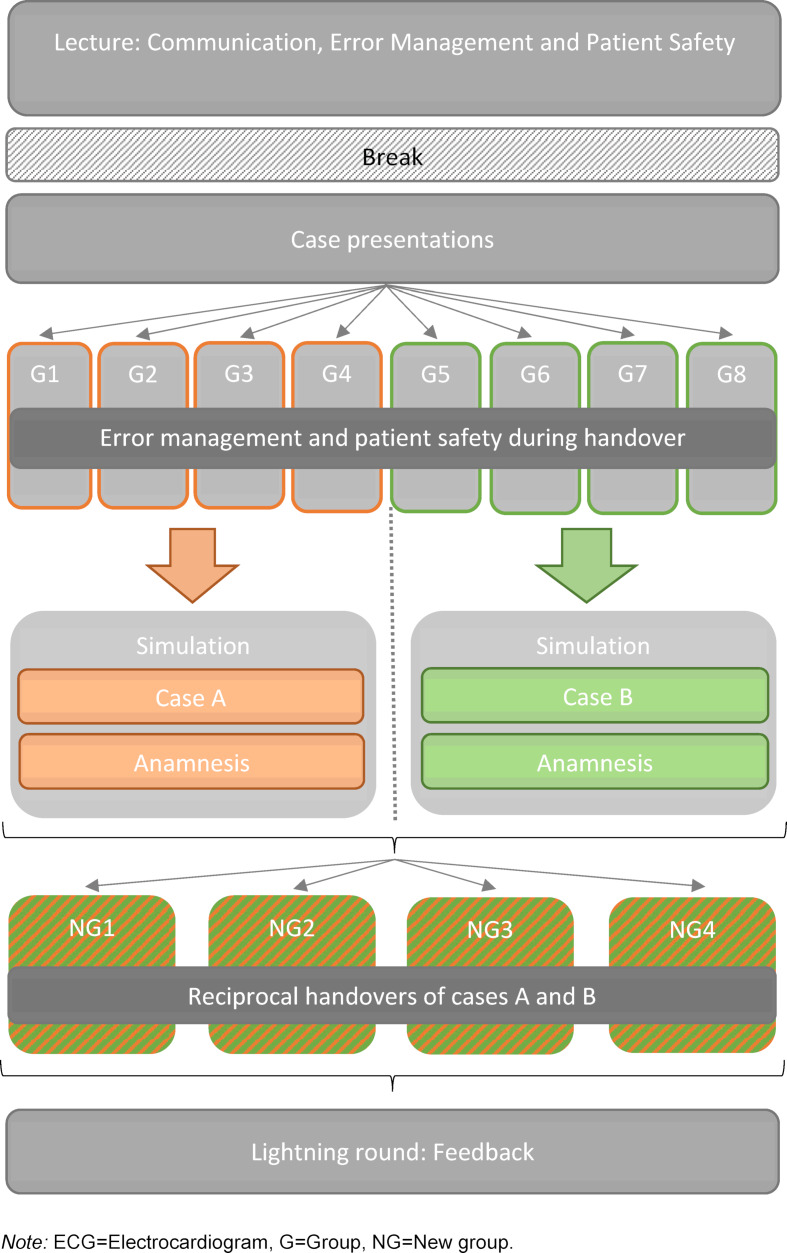
Course sequence and content-related design of the digital preparatory course for the clinical traineeship

At the end there was a lighting round for feedback in the big group with all attendees. An illustration of the entire course sequence is presented in figure 1 (Fig. 1).

#### 2.2. Evaluation

The evaluation took place in July 2020. The attendees were surveyed anonymously using an online questionnaire before starting and after completing the elective preparatory course to self-assess their knowledge and confidence in performing practical telemedical skills. The online questionnaire contained items developed by the authors (see attachment 6 and attachment 7 ).

The course was held on three days with a maximum of 40 attendees per day. The students who completed the preparatory course satisfied the basic inclusion criterion. No exclusion criteria were defined.

#### 2.3. Sample

**Figure 2:**
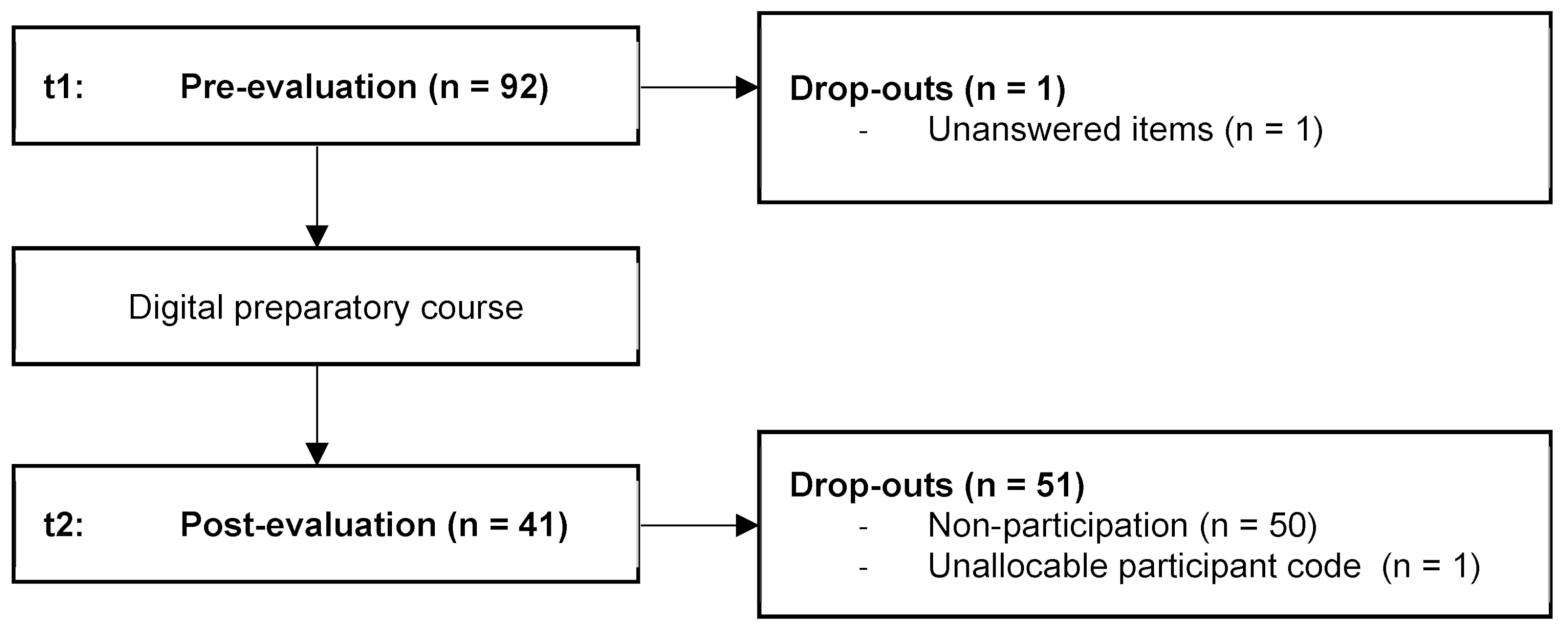
Flowchart

A total of 93 people participated in the survey conducted at timepoint t1 (pre measurement). One person had to be excluded from the analysis due to unanswered items so that the total sample is N=92 (21.6 years±3.32; female 80.4%, male 18.5%; other 1.1%) and corresponds to a response rate of 76.67% with a total of 120 course attendees. A total of 42 people participated in the survey after completion of the preparatory course (t2). One dataset had to be excluded from further analysis due to an unattributable personal code, which is why the sample size is reduced to n=41 (see figure 2 (Fig. 2)).

Data analysis was carried out using IBM SPSS Statistics Version 26 (IBM Corp., Armonk, NY, USA). The evaluation of the digital preparatory course included both the descriptive statistics for the overall evaluation and the evaluation of the course segment into which the SPs were integrated. This entails giving the mean values and standard deviations. The pre-post data were also compared in terms of subjective confidence concerning knowledge-based skills and the performance of clinical skills before and after completing the digital preparatory course and then analyzed for significant changes. The Shapiro-Wilk tests showed significant deviations from the normal distribution not only for the pre and post data but also for their differences (all *p*<.05). For this reason, the pre-post comparisons were calculated using Wilcoxon's signed-rank test.

#### 2.4. Ethics

Within the scope of this study only anonymous data was collected by questionnaire making it impossible to match a specific person to the data. The participants were informed in detail about the purpose of the study in advance. This study was approved by the independent ethics commission at RWTH Aachen University (EC no. 21-138).

## 3. Results

### Evaluation

#### 1. Descriptive statistics

A total of 31.0% of the respondents rated the course as “very good” and 52.4% as “good”.

The descriptive statistics regarding acquired abilities and skills associated with the simulated patients are given in table 1 (Tab. 1).

##### 2. Pre-Post analysis of subjective confidence

For all of the tested items, the results of Wilcoxon's test for dependent samples showed highly significant increases in perceived confidence after taking the preparatory course (see table 2 (Tab. 2)). These remained so even after applying Bonferroni correction for multiple test comparisons (adjusted threshold value p*=0.01).

## 4. Discussion

The present study shows that telemedicine modules integrated into a digital preparatory course positively contribute to students’ subjective confidence regarding knowledge and confidence in performing practical telemedical skills. In particular, this study makes it clear that professional communication between a doctor and patient, digital case history taking, and handover techniques can be learned through telemedical course content.

Telemedicine has very suddenly gained importance (not just) in Germany as a result of the COVID-19 pandemic. It is to be expected that this development will continue to progress even after the pandemic is over, with considerable potential specifically in regard to the shortage of skilled workers and optimizing resource use [12], [13]. Remarkably, patients often had a more positive attitude toward telemedicine than physicians [14], who often feared that the quality of medical advice would suffer or the doctor's appointment could become too impersonal [15]. The results of the present study suggest that these kinds of apprehensions are not justified. The evaluation of the course segment with simulated patients shows that the students were subjectively able to establish a solid doctor-patient relationship also in a digital setting (mean 4.05 of 6). Doubts about telemedicine frequently seem to be based on a lack of experience. It has been shown that physicians who already had experience with the uses and benefits of telemedicine gave it a distinctly higher rating in an overall evaluation [16] and, in particular, recognized its usefulness much more clearly than others did [17]. Given this, it appears that much more important to expose future physicians to telemedicine and counteract potential reservations they might have through practical experience.

When developing future telemedical course content, it is important to note that this field is especially subject to rapid change due to the fast-paced advances in technology. Specific course content should be checked regularly to be sure that it is current and revised or replaced, if necessary. Likewise, telemedicine curricula should include training for future users in the different areas of application (hospital, emergency physician, general practitioner) to deal with the ethical, legal and regulatory implications of telemedicine [18]. Furthermore, it is important to illustrate the conceptual integration of technology using examples and thus open up new areas for telemedicine. This is not to say that theory chases practice, but rather that this must be actively designed and structured.

This course has the characteristics of a model course with its novel combination of digital preparation (Moodle), theoretical teaching of basic knowledge, and practical exercises including simulation. The organizational sequence of the course follows the principles of Bloom and Miller [https://www.bloomstaxonomy.net/], [19], which, in this form, poses an innovation in the digital setting.

Technical factors could be a challenge when implementing telemedicine courses. Courses (like the one presented here) which require the assignment of attendees to breakout rooms, connections to instructors and SPs, and changes to the group sizes mid-way through will need careful preparation and good technical support, including during the course. Here it is essential to consider the unique link between (digital) teaching format and (digital) learning content for these courses. Technical difficulties when conducting a course session could have especially negative impacts since such difficulties also hinder the learning process and could reinforce potential doubts about telemedicine. As a consequence, ensuring that courses go smoothly is very strongly advised.

### Limitations

Since this study analyzes data on self-assessment by students, the strength of the conclusions that can be drawn from the results is subject to certain limitations. Based on these results, no statements can be made about the students’ actual gain in knowledge nor their objective performance of practical clinical skills. Moreover, no conclusions can be drawn about a direct comparison of the digital course with the classroom-based course.

Another limitation affects the relatively high drop-out rate among the survey participants of over 50% between the pre- and post-test. The reason for this can only be speculated; it is possible that the participants were no longer sufficiently motivated to fill out another evaluation after completing the course. There was no indication that this drop-out rate could stand in a systematic relationship to the evaluation of the course, but this obviously cannot be ruled out and therefore must be taken into consideration when interpreting the results. A further limitation is the unequal distribution of the participants in regard to gender. The percentage of female participants was over 80%; a bias in the results due the unequal gender distribution cannot be ruled out. However, it must be kept in mind that the actual gender distribution among medical students in Germany does reflect a high percentage of women (2/3 female to 1/3 male) [20].

## 5. Conclusion

Telemedical modules, integrated into the existing medical curriculum based on the example of a digital clinical traineeship preparatory course, increase students’ subjective confidence regarding knowledge and confidence in performing practical telemedical skills. Early implementation of the modules reduced uncertainty and prejudice and strengthened experiences for the purpose of preparing future physicians to meet the demands of telemedicine in routine medical practice. This is of critical importance in view of the increasing digitalization in medicine. 

## Competing interests

The authors declare that they have no competing interests. 

## Supplementary Material

Checklists

Participant instructions case examples 1-5

Instructor information case example A

Instructor information case example B

Participant instructions case examples A and B

Pre-evaluation

Post-evaluation

## Figures and Tables

**Table 1:**
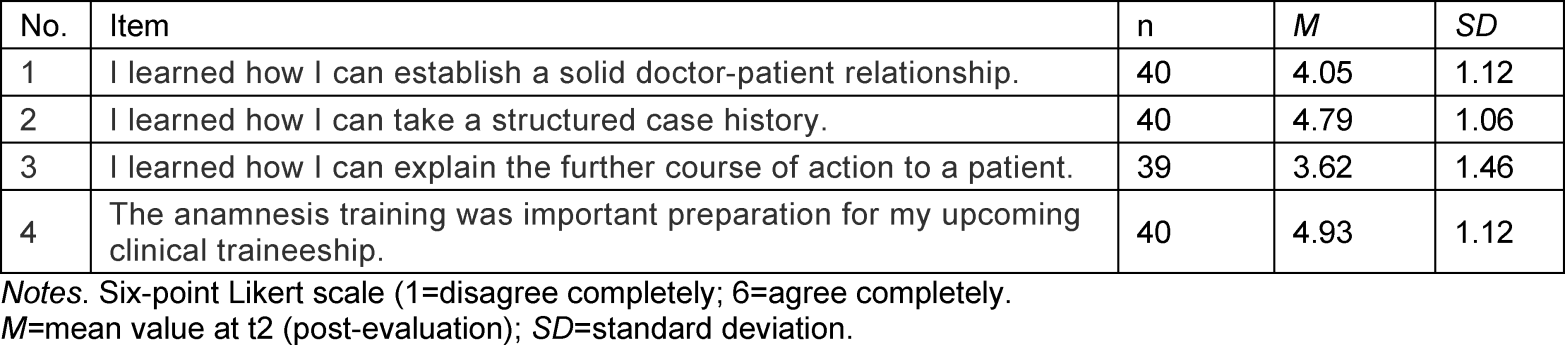
Descriptive statistics for the course segment with simulated patients

**Table 2:**
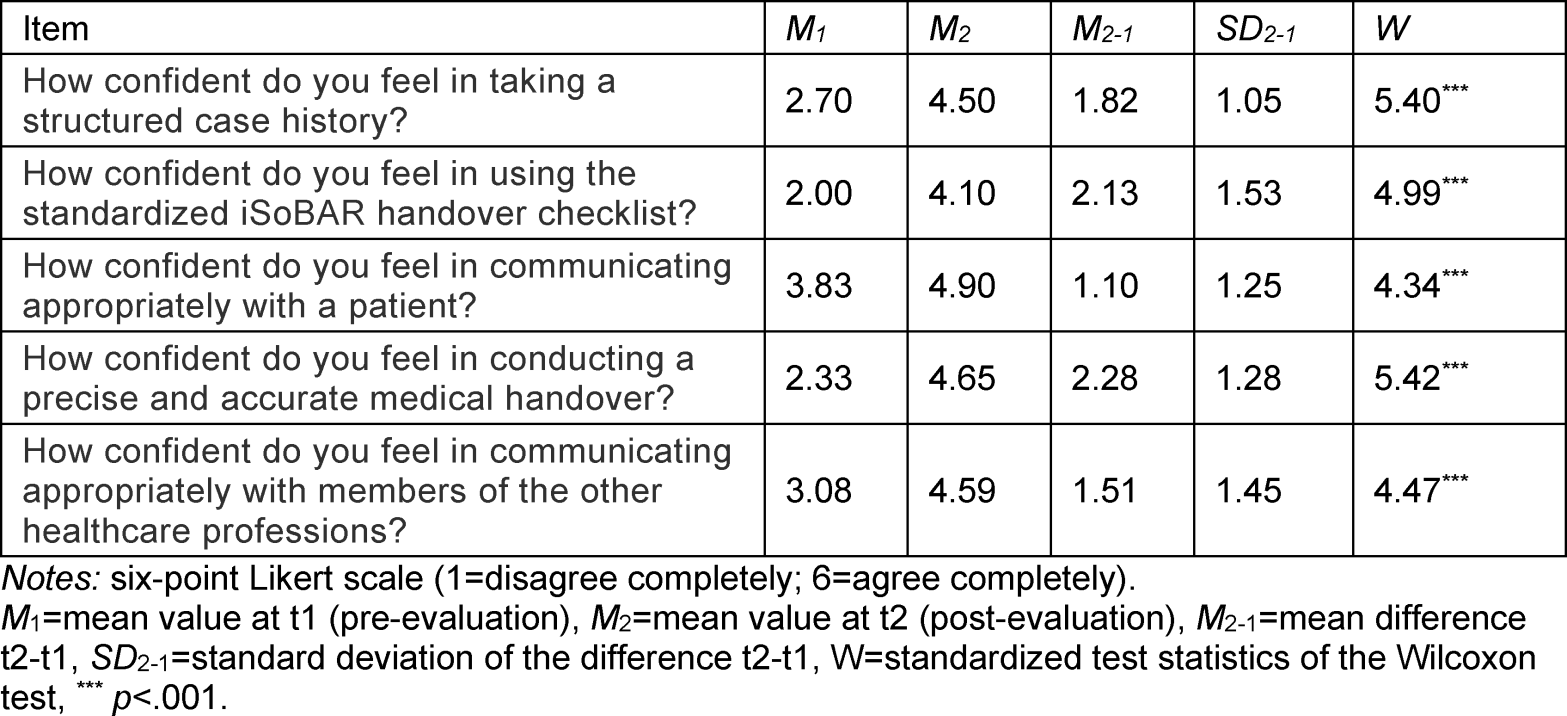
Results of the Wilcoxon test for paired samples regarding the self-assessed confidence in performing practical telemedical skills.
