# Supplementary material for: Telemedicine in medical education: An example of a digital preparatory course for the clinical traineeship – a pre-post comparison
Source: GMS J Med Educ. 2022 Sep 15;39(4):Doc46. doi: 10.3205/zma001567 (PMC9585416; doi:10.3205/zma001567)
Supplement: Checklists [file JME-39-46-s-001.pdf]

Attachment 1: Checklists

Attachment 1 to Vogt L, Schmidt M, Follmann A, Lenes A, Klasen M, Sopka S. *Telemedicine in medical education: An example of a digital preparatory course for the clinical traineeship – a pre-post comparison*. GMS J Med Educ. 2022;39(4):Doc46. DOI: 10.3205/zma001567

ABCDE

**First impression**	**Location / Safety / Situation**
⇒ Responsive?<br>⇒ Breathing / pulse?<br>⇒ Significant bleeding visible?	⇒ What, where, how, how many patients? Trapped?<br>⇒ Your own safety<br>⇒ Kinematics? Weather? Nearest trauma center?

A

Airways / Cervical Spine

- Airways unobstructed? Fixate neck!
- Inspect mouth / throat
- Reposition? Foreign objects?

B

Breathing / Ventilation

- Breathing rate / Respiratory depth (sufficient?)
- Auscultation
- Chest movements & stability, cervical veins?

C

Circulation

- Heavy bleeding visible / suspected?
- Pulse, peripheral / central
- Pulse quality / frequency
- Skin temperature & color / CRT

D

Disability / Neurological Deficits

- GCS evaluation
- Pupils responsive?

E

Environment / Exposure

- Examine (potential sources of blood?)
- Review findings
- Retain warmth

Critical Patient?

⇒ Emergency rescue?
⇒ Time management – Evaluate transport priority
⇒ Re-evaluate ABCDE
⇒ Check indications for full-body immobilization

See ATLS (Advanced Trauma Life Support)

ISBAR

**I**	**Identify – Who are You and Who is the Patient?**<br>⇒ Your name, role in providing healthcare<br>⇒ Patient: name, age/DOB, sex, ID no., room no.<br>⇒ Admission date/reason for hospitalization
**S**	**Situation – What is going on with the Patient?**<br>⇒ Reason for hospital admission<br>⇒ Main diagnosis/problem(s)<br>⇒ e.g., ABCDE checklist
**B**	**Background – What is the Clinical Background / Context?**<br>⇒ Relevant pre-existing disease<br>⇒ Allergies<br>⇒ Social & psychiatric background<br>⇒ Treatment course<br>⇒ Medications<br>⇒ Current tests
**A**	**Assessment/Evaluation – What could be the Problem?**<br>⇒ Most recent clinical findings and tests<br>⇒ Has the current problem been discussed?
**R**	**Recommendation – What would you suggest?**<br>⇒ Necessary measures<br>⇒ Additional exams or tests needed? Consultations?
	**Read-back – Confirm handover information**<br>⇒ Confirm understanding / summarize handover information
	**Risk – What are possible risks?**<br>⇒ Re-confirm understanding / summarize handover information<br>⇒ "Safety pause"

See also: Safety and Quality Unit, Department of Health: www. sahealth.sa.gov.au/safetyandquality

MIST

Patient

FIRST: Brief, focused and proper handover

SECOND: Place patient on the shock room table

Handover to the shock room team communicates:

M

Mechanics of the Accident

⇒ Fall from what height; motorcycle accident; trapped, etc.

I

Identified Injuries

- Which injuries are known?

S

Symptoms and Signs (ABCDE checklist)

- A Airways kept unobstructed while protecting cervical spine
- B Breathing and sufficient ventilation
- C Circulation: stabilize and check for hemorrhaging
- D Disability: neurologic status
- E Exposure/Environmental control

T

Therapy and transport time, kind of transport

Age of patient

See Schockraumübergabe der Notärzte im Rettungsdienst Aachen e.V. & Uniklinik RWTH Aachen

See ATLS (Advanced Trauma Life Support)

SAMPLER

S — Symptoms

Pain / Symptoms:
- Start, location, progression, length of time, factors
- Exacerbation, deterioration
- Quality, characteristics , intensity

A — Allergies
- Allergy documenation
- Critical before giving emergency medications!

M — Medication
- Length of medication
- New medication
- Regular schedule; when was last time?

P — Past medical history
- Current disease
- Pre-existing conditions, surgeries, pregnancy?
- Diabetic, Marcumar, defibrillator/pace-maker? Prenatal chart?

L — Last oral intake
- What?
- How much?
- Fluid? Solid?

E — Event
- Events of accident or acute sickness; mechanics of the accident, circumstances, actions prior to start of symptoms

R — Risk
- Risk factors?
- Hypertension? CHD? Diabetes?
