# Supplementary material for: Telemedicine in medical education: An example of a digital preparatory course for the clinical traineeship – a pre-post comparison
Source: GMS J Med Educ. 2022 Sep 15;39(4):Doc46. doi: 10.3205/zma001567 (PMC9585416; doi:10.3205/zma001567)
Supplement: Participant instructions case examples 1-5 [file JME-39-46-s-002.pdf]

Attachment 2: Participant instructions case examples 1-5

Attachment 2 to Vogt L, Schmidt M, Follmann A, Lenes A, Klasen M, Sopka S. *Telemedicine in medical education: An example of a digital preparatory course for the clinical traineeship – a pre-post comparison*. GMS J Med Educ. 2022;39(4):Doc46. DOI: 10.3205/zma001567

Anamnesis and Handover Training Part 1

Your assignment:

Each student will work on **one** case from cases A1-A5. You have 30 minutes to extract all of the relevant information from the case vignette and any additional information (cases A1-A3) and use it to structure a handover.

After the 30 minutes have passed, you will hand the case over to your group (breakout room). Please try to present all of the relevant information in a structured manner. Different checklists are available in Moodle to serve as aids, which you can use but are not required to. Each group is assigned to an instructor who will give you feedback and assistance with doing the assignment.

Case vignettes Part 1 2

... Case 1: Traffic Accident (Müller) 2
... Case 2: Transfer to Stroke Unit (Petz) 3
... Case 3: Transfer from the ER (Lehmann) 4
... Case 4: Referral to Cardiologist (Schulze) 5
... Case 5: Convulsing Infant (Meier) 7

Example case 1: Traffic Accident

Patient: Dieter Müller, 57 years old

You have been called as the emergency physician to a car accident on the A4 just past the Aachen turnpike. A car veered from its lane and crashed into the guardrail at a speed of about 80km/h.

The front and right side of the vehicle are smashed in. Upon arrival, the only person in the car is the driver. He responds to painful stimulus only with random defensive movements. A snoring-like breathing sound is clearly audible. You recognize bruising on the left and right sides of the chest as well as on the upper abdominal area. Rapid extrication is performed by the fire fighters.

During the subsequent examination in the ambulance, swelling is noticed in the right thigh. Pupils are isocor and react to light before and after intubation. Lacerations on the forehead and left side of the calvaria indicate traumatic brain injury. The patient was fully immobilized by the emergency responders with a Stifneck on a spineboard and the shock room at the Aachen University Hospital has been alerted and is expecting the patient.

On the way to the hospital, you fill out the documentation and are ready for the handover to the team in the shock room.

Example case 2: Transfer to the Stroke Unit

Patient: Dorothea Petz, 71 years old

You are the ward physician in the hospital's ophthalmic department. One of your patients, Mrs. Petz, was admitted to the main ward the day before yesterday for elective amotio surgery and operated on yesterday. The surgery was performed under general anesthesia. Mrs. Petz was moved from the recovery room after being monitored for one and a half hours and transferred back to your ward in the ophthalmic department.

Mrs. Petz has various pre-existing conditions and takes several medications, which have already been prescribed by her general practitioner, and her medication schedule was noted when she was admitted.

You are called to Mrs. Petz's bedside by the ward nurses for an emergency: Mrs. Petz has fallen from her chair and is not moving.

As you enter the room, the patient is lying on the floor next to the chair with a fixed stare.

You record the following vitals:

- RR 150/90
- HF 89/min., arrhythmic
- SpO2 95%
- AF 18/min.

The patient opens her eyes when spoken to, but only makes incomprehensible sounds. The corners of her mouth are pointing downward. When asked to move, Mrs. Petz moves only somewhat on her right side. You shine a penlight in the patient's eyes: pupils are equal, round and react to light bilaterally.

After your first examination of the patient, you suspect a stroke. Before you can phone the neurologist on duty in the stroke unit, you look once more at the medication schedule the patient brought with her.

The patient is regularly taking Marcumar for atrial fibrillation, the administration of which was suspended by the general practitioner prior to hospital admission--and the same for ASS.

After you have inspected the medication schedule in light of the relevant pre-existing conditions, you alert the stroke unit by telephone and organize the transfer to the shock room.

Example case 3: Transfer from the ER

Patient: Anne Lehmann, 32 years old

The patient Anne Lehmann was admitted in the ER. Now she is being transferred from the ER to you on your ward with only the handwritten admission form. Her blood sugar level is currently at 280 mg/dl.

You are new to the profession and new on the ward. So you consult by telephone with your supervising senior physician about what action to take.

Example case 4: Referral to Cardiologist (page 1/2)

Patient: Jürgen Schulze, 53 years old

Mr. Schulze, a longtime neighbor of your parents, presents with unspecific, recurring chest pain at your general practice. After you have completed your diagnostics, you arrange for an appointment with a trusted cardiologist. You present the patient case in advance on the telephone in a physician-to-physician consultation.

Example case 4: Referral to Cardiologist (page 2/2)

Excerpt from the patient's medical chart:

Anamnesis:
Mr. Schulze is a 53-year-old in a normal general and nutritional condition (181cm, 73kg). The patient came in today with recurring, unspecific chest pain which has existed for about 8-10 weeks and is described as a tight pain radiating, in part, to the back. The pain occurs sometimes daily, sometimes in a weekly rhythm, always during the daytime and during physical exertion and is self-limiting once the exertion is over. Patient states he has had back pain for years. No problems sleeping at night. Up until about three years ago, Mr. Schulze exercised regularly (running, cycling), but has stopped for work-related reasons. As an office worker, he sits mainly while working, but is quite challenged in terms of his work. Elevated blood lipid levels are known to be on his paternal side; there has also been a cardiac infarct. His mother suffers high blood pressure and very late-onset diabetes mellitus. Mr. Schulz is not currently taking any medications.

Physical exam:
The physical exam shows situationally elevated blood pressure (RR 164/92mmHg) with a heart rate of 72 beats per minute. Auscultation and percussion findings are normal; clear and regular heart sounds. The paravertebral musculature is distinctly tight, vertebral blockage at TH1/2. The rest of the exam is without pathological findings.

Measure taken at the practice:
An ECG was done; the blockage was released through chiropractic manipulations of the spine. Soft tissue techniques also applied to relax the musculature. The patient was then shown physical exercises to do at home. A sheet with all of the instructions was handed out.

The ECG showed a repolarization abnormality (T-wave negativity in aVR, V2-5). A stress ECG was done at the practice in response. This was without problem up to 125 Watts; discrete ST depressions were seen around 0.12mV in lead V6. Normal blood pressure regulation under stress. The exam was stopped prematurely due to peripheral exhaustion.

The results and the suspected diagnosis of coronary heart disease with angina pectoris under strain and the diagnostic plan of action for known risk profile was discussed in detail with the patient. Drug therapy was initiated (anti-hyperintensive, statin). An appointment with a cardiologist will be made for further diagnostics and therapy.

Example case 5: Convulsing Infant (page 1/3)

Patient: Lea Meier, 6 months old

The mother of the six-month-old infant called emergency services because the baby was having a seizure. The alarmed emergency physician reported that upon arrival at the home, the girl was convulsing perioral and in the extremities; showed cyanotic skin color and rolled her eyes upwards to the right. The seizure ceased after rectally administering 5 mg of diazepam. The infant with the mother was then brought by ambulance to the hospital ER where you are responsible for pediatric care of young infants.

The mother reports that Lea was completely healthy up until that morning, but she threw up her lunch two hours after eating. She also felt hot to the touch. The mother then gave her an antipyretic suppository (paracetamol 125 mg). Several hours later at the next mealtime, the mother went into the bedroom and found Lea convulsing in her crib. While the case history was being taken, the mother remembered that the child had not defecated since the day before; there was no diarrhea.

Excerpt from the patient's medical chart:

Anamnesis: Lea's birth was full term and spontaneous; birth weight 3600 g and length 52 cm. Lea is the family's third child. Her siblings are healthy. Her development has been fully normal and age-appropriate. Lea weighs 6800g with a length of 67cm. No prior seizures have been seen in Lea. No known family history of disease or seizures. Three weeks ago Lea belatedly received the first 6-fold immunization; no reaction to the vaccine was observed. No infectious diseases circulating in the area. No known allergies to date.

Physical exam: At hospital admission the infant was in a reduced state of health; she responded to external stimulus, but did not open her eyes and resisted only half-heartedly to the initial measures, such as placement of an i.v. line and taking blood samples. She fell back asleep immediately after. Respiration is accelerated at 60/min, auscultation found neither dullness nor rales. Tachycardia at around 190/min. Extremities are cool; skin appears marbled; rectal temperature of 39.1 °C. Abdomen is soft; no hepatosplenomegaly or noticeable resistance is palpable. ENT: no signs of acute infection. Fontanelle is positioned normally, not bulging. Lea is very sensitive to touch and startles easily. Since there is reason to suspect an infection but this is not immediately confirmable during the first exam, blood and urine samples are taken for further diagnostic tests.

Example case 5: Convulsing Infant (page 2/3)

Findings:

a) Lab results:

Hb	148 g/L
Thrombozyten	82/nL
Leukozyten	2800/µL

Differenzialblutbild:

Promyelozyten	2 %
Metamyelozyten	2 %
stabkernige Neutrophile	12 %
segmentkernige Neutrophile	15 %
Lymphozyten	59 %
Monozyten	10 %
C-reaktives Protein	1,8 mg/dL
Glukose	165 mg/dL
Gesamt-Eiweiß	67 g/L
Natrium	138 mmol/L
Kalium	4,9 mmol/L
Calcium	2,2 mmol/L

b) Blood gas analysis:

pH	7.10
Base excess	-15.5 mmol/L
pCO2	28 mmHg
pO2	45 mmHg

c) Urine status:

No pathological findings

d) The first results of the liquor test are available after 45 minutes:

Leukocytes:	240/µL (reference range: < 4/µ/L),
Protein:	45 mg/dL (reference range: < 45 mg/dL)
Glucose:	25 mg/dL (reference range: 38-65 mg/dL)

The results of the liquor cultures are expected in 2-3 days.

Example case 5: Convulsing Infant (page 3/3)

As Lea is being changed by her mother and a nurse, the nurse reports to you that she noticed distinct skin changes on Lea which were not there an hour before at the time of admission (see photo).

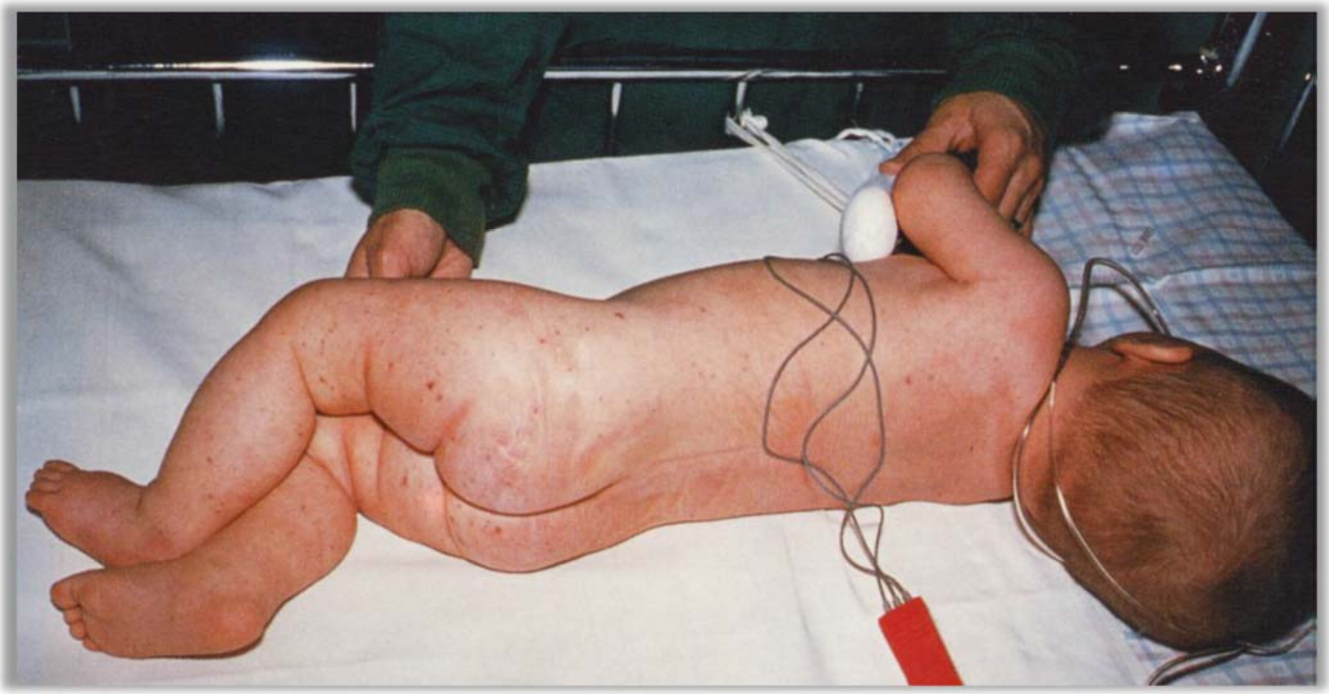

You order the immediate start of antibiotic therapy and transfer Lea to the pediatric intensive care unit. There you hand the case over to the attending intensive care physician.
