# Supplementary material for: Telemedicine in medical education: An example of a digital preparatory course for the clinical traineeship – a pre-post comparison
Source: GMS J Med Educ. 2022 Sep 15;39(4):Doc46. doi: 10.3205/zma001567 (PMC9585416; doi:10.3205/zma001567)
Supplement: Instructor information case example A [file JME-39-46-s-003.pdf]

Attachment 3: Instructor information case example A

Attachment 3 to Vogt L, Schmidt M, Follmann A, Lenes A, Klasen M, Sopka S. *Telemedicine in medical education: An example of a digital preparatory course for the clinical traineeship – a pre-post comparison*. GMS J Med Educ. 2022;39(4):Doc46. DOI: 10.3205/zma001567

Example case A: Back Pain, Rieger (page 1/2)

Patient: Rieger (Actor's age and gender)

The medical student is a clinical trainee in the ER of the Aachen University Hospital. A patient calls during telephone consultation hours and the on-duty assistant physician asks the student to take a detailed case history via video conference. Following this, the case needs to be handed over to the on-duty assistant physician (the group).

Biographical background

Each simulated patient uses his/her own personal biography. Their true medical history, including any diseases or surgeries, should be left out of the considerations.

Backstory for actors (symptoms)

- Yesterday afternoon you worked in the garden for several hours. You were bending over often and squatting for long periods of time as you worked. Each time when you stood up you had wrenching pain in your lower back. This is not entirely new to you; you have noticed it for months now.
- Shortly before you finished working in the garden, you picked up a big pot and twisted your upper body to the left. As you did this you experienced a strong stabbing pain in the lumbar spine that radiated out into your left leg. You set the pot down immediately and laid down on the sofa.
- Your partner rubbed an arthritis salve on you and gave you heating pads. It got better after that. In the night you just had slight pain overall; the stabbing pain that spread into your leg is gone. Still, you fear that it will come back when you exert yourself.

When questioned further, the actor shares this information:

(S)AMPLER

Age of actor < 30 years	Age of actor 30 years
• A: pollen allergy/hay fever in summer<br>• M: Loratadine 10mg Tab. p.o. 1-0-0-0<br>• P: blank<br>• L: granola, coffee with milk for breakfast<br>• E: see below<br>• R: blank	• A: Novalgin allergy**<br>• M: ramipril 5mg Tab. p.o. 1-0-0-0<br>• P: art. hypertension (presently normotensive)<br>• L: milk soup, with black coffee<br>• E: see below<br>• R: blank

**First sign: red and itchy rash on leg and chest after about 3 hours

Example case A: Back Pain, Rieger (page 2/2)

When questioned further, the actor shares this information (continued):

- No known pre-existing conditions/diagnoses regarding the back
- No numbness in leg
- No neurological deficits
- No problems urinating
- Previous OPs: no surgeries in the area of the back, appendectomy as child

Current symptoms acted out by the actor in this scenario

- You assume a slightly protective posture in your gait (for fear of renewed pain).
- You feel a pulling sensation in your lower back on both sides, increased pain occurs above all when you sit down, which is nearly impossible for you. Once on the chair, you assume a protective posture (cautiously sitting down on the edge of the chair, hands supporting you as you sit up straight, (possibly sitting down on only half of your bottom).

Learning objectives

- Establishing a solid doctor-patient relationship
- Taking a structured case history
- Developing and explaining/discussing the further medical treatment (communicative focus)
- Structuring a handover

Example case A: Patient Rieger

You are doing a clinical traineeship in the ER at the Aachen University Hospital. It is 10:00 a.m.

The assistant physician on duty asks you to take a detailed case history via video conference with a patient.

Your task: Record a current case history and consider what actions to take next. Then hand the case over in a structured manner with all of the relevant information to the physician on duty.
