# Supplementary material for: Telemedicine in medical education: An example of a digital preparatory course for the clinical traineeship – a pre-post comparison
Source: GMS J Med Educ. 2022 Sep 15;39(4):Doc46. doi: 10.3205/zma001567 (PMC9585416; doi:10.3205/zma001567)
Supplement: Instructor information case example B [file JME-39-46-s-004.pdf]

Attachment 4: Instructor information case example B

Attachment 4 to Vogt L, Schmidt M, Follmann A, Lenes A, Klasen M, Sopka S. *Telemedicine in medical education: An example of a digital preparatory course for the clinical traineeship – a pre-post comparison*. GMS J Med Educ. 2022;39(4):Doc46. DOI: 10.3205/zma001567

Example case B: Vomiting & Diarrhea, Metzler (page 1/2)

Patient: Metzler (Actor's age and gender)

The medical student is a clinical trainee at a general medical practice in the center of Aachen. A thorough case history needs to be taken via video conference and then the case needs to be handed over to the on-duty assistant physician.

Biographical background

Each simulated patient uses his/her own personal biography. Their true medical history, including any diseases or surgeries, should be left out of the considerations.

Backstory for actors (symptoms)

- Since yesterday around 2 p.m., you have had increasing feelings of nausea since eating a salad with mozzarella. Four hours later you vomited for the first time, and threw up part of that meal.
- By 8 p.m. you had thrown up six more times and in the end there was only white foamy slime coming out. You cannot eat or drink because of the overwhelming nausea.
- From 9 p.m. onward you also had stomach cramps; your entire abdominal area hurt, sometimes more, sometimes less.
- From 10 p.m. onward you had diarrhea, too, that kept you running to the toilet. This was watery and brown.
- The pain was less right after using the toilet but came back every time.
- You last went to the toilet at 4 a.m. Since then you do not have diarrhea any more, the pain is much less. You still have pain all over your abdomen.

When questioned further, the actor shares this information:

(S)AMPLER

Age of actor < 30 Jahre	Age of actor > 30 Jahre
• A: iodine allergy**<br>• M: simvastatin 40mg Tab. p.o. 0-0-1-0<br>• P: dyslipidemia, appendectomy as child<br>• L: mozzarella salad, yesterday 2 p.m.<br>• E: see below<br>• R: blank	• A: hay fever in summer<br>• M: salbutamol inhaler, as needed<br>• P: allergic asthma, appendectomy as child<br>• L: mozzarella salad, yesterday 2 p.m.<br>• E: see below<br>• R: blank

**Skin rash after administration of contrast agent at the radiologist's, went away within a few hours with medication; it involved a very itchy, red rash all over the body; no problems with circulatory system occurred.

Example case B: Vomiting & Diarrhea, Metzler (page 2/2)

When questioned further, the actor shares the following information (continued):

- No fever.
- No known pre-existing conditions in the intestine.

Current symptoms

- Nausea is gone since the last episode of vomiting around 2 a.m. In general, you feel drained; you still have moments of extreme dizziness, you feel very weak and shaky.
- The pain continues, your entire stomach is "sore on the inside."
- You now want to be sure that it is nothing serious.

Learning objectives

- Establishing a solid doctor-patient relationship
- Taking a structured case history
- Developing and explaining/discussing the further medical treatment (communicative focus)
- Structuring a handover

Example case B: Patient Metzler

You are doing a clinical traineeship at a general practitioner's office in the center of Aachen. It is 8:00 a.m., the practice has just opened. A patient needing medical help is already on hold, waiting for a telephone/video appointment.

The general practitioner asks you to take the patient's case history via video conference.

Record a current case history and consider what actions to take next. Then hand the case over in a structured manner with all of the relevant information to the attending physician.
