# Supplementary material for: Telemedicine in medical education: An example of a digital preparatory course for the clinical traineeship – a pre-post comparison
Source: GMS J Med Educ. 2022 Sep 15;39(4):Doc46. doi: 10.3205/zma001567 (PMC9585416; doi:10.3205/zma001567)
Supplement: Participant instructions case examples A and B [file JME-39-46-s-005.pdf]

# Attachment 5: Participant instructions case examples A and B

Attachment 5 to Vogt L, Schmidt M, Follmann A, Lenes A, Klasen M, Sopka S. *Telemedicine in medical education: An example of a digital preparatory course for the clinical traineeship – a pre-post comparison*. GMS J Med Educ. 2022;39(4):Doc46. DOI: 10.3205/zma001567

# Anamnesis and Handover Training Part 2

**Your assignment:**

You are to work on either case A (Groups G1, G2, G3, G4) **or** case B (Groups G5, G6, G7, G8)

For the first part you will take a structured case history--as you have already learned to do in your previous training. Your patient is connected to you by video for this appointment. Please pay attention to the structure of your anamnesis and try to record all of the relevant information. The anamnesis can be conducted individually or as a group.

In the second part you will hand your case over in a structured manner to another small group. The groups will be formed automatically by the Zoom moderator.

# Example case A: Patient Rieger

You are doing a clinical traineeship in the ER at the Aachen University Hospital. It is 10:00 a.m.

The assistant physician on duty asks you to take a detailed case history via video conference with a patient.

Your task: Record a current case history and consider what actions to take next. Then hand the case over in a structured manner with all of the relevant information to the physician on duty.

# Example case B: Patient Metzler

You are doing a clinical traineeship at a general practitioner's office in the center of Aachen. It is 8:00 a.m., the practice has just opened. A patient needing medical help is already on hold, waiting for a telephone/video appointment.

The general practitioner asks you to take the patient's case history via video conference.

Record a current case history and consider what actions to take next. Then hand the case over in a structured manner with all of the relevant information to the attending physician.
