# Supplementary material for: Telemedicine in medical education: An example of a digital preparatory course for the clinical traineeship – a pre-post comparison
Source: GMS J Med Educ. 2022 Sep 15;39(4):Doc46. doi: 10.3205/zma001567 (PMC9585416; doi:10.3205/zma001567)
Supplement: Pre-evaluation [file JME-39-46-s-006.pdf]

Attachment 6: Pre-evaluation

Attachment 6 to Vogt L, Schmidt M, Follmann A, Lenes A, Klasen M, Sopka S. *Telemedicine in medical education: An example of a digital preparatory course for the clinical traineeship – a pre-post comparison*. GMS J Med Educ. 2022;39(4):Doc46. DOI: 10.3205/zma001567

Clinical Traineeship Preparatory Course - PRE

Dear students,

Thank you for taking the time to fill out this questionnaire.
The aim is to evaluate our Clinical Traineeship Preparatory Course at Aixtra and to adapt the teaching to the needs of the students.
This survey will take about 10 minutes.
Participation is voluntary and anonymous. No one will be able to match you with your answers.
If you have any questions, we will be happy to answer them. Thank you for your participation!

AIXTRA Team

Personal Code

Please enter your **personal code** according to the following instructions:

First letter of your mother's first name
First letter of your father's first name
Your mother's day of birth
Your father's day of birth

Example:
Mother's first name: Anna => A
Father's first name: Max => M
Mother's day of birth: March 5th => 05
Father's day of birth: November 12th => 12
Example code: AM0512

Personal code

Survey generated with LamaPoll

Course date

Please select the date of your course:

Date

Age

Please enter you age here:

Age

Gender

- ◯ Female
- ◯ Male
- ◯ Other

Professional Experience

Previous work experience in

- ◯ Nursing
- ◯ Emergency Medical Services
- ◯ Neither

Semester Level

☐ 1st ☐ 2nd ☐ 3rd ☐ 4th ☐ 5th ☐ 6th ☐ 7th ☐ 8th ☐ 9th ☐ 10th ☐ 11th ☐ 12th

Survey generated with
LamaPoll

Previous Clerkships

	Yes	No
I have already attended a clinical traineeship preparatory course.	◯	◯
I have already completed one or more clinical traineeships.	◯	◯

Survey generated with
LamaPoll

In which subject or subjects have you already completed a clinical traineeship?

- [ ] Surgery
- [ ] Internal Medicine
- [ ] General Practice
- [ ] Gynecology, Urology
- [ ] Pediatrics
- [ ] Neurology
- [ ] Anesthesiology
- [ ] Orthopedics/Emergency Surgery
- [ ] Psychiatry
- [ ] Psychosomatics
- [ ] Radiology
- [ ] Nuclear Medicine
- [ ] Ophthalmology
- [ ] Otorhinolaryngology
- [ ] Dermatology
- [ ] Anatomy
- [ ] Occupational Medicine
- [ ] Environmental Medicine
- [ ] Human Genetics
- [ ] Microbiology and Virology
- [ ] Pathology
- [ ]

Survey generated with
LamaPoll

- [ ] Pharmacology
- [ ] Phoniatrics
- [ ] Forensic Medicine
- [ ] Other

Survey generated with
LamaPoll

In which subject areas do you wish to complete your (other) clinical traineeships?

- [ ] Surgery
- [ ] Internal Medicine
- [ ] General Practice
- [ ] Gynecology
- [ ] Urology
- [ ] Pediatrics
- [ ] Neurology
- [ ] Anesthesiology
- [ ] Orthopedics/Emergency Surgery
- [ ] Psychiatry
- [ ] Psychosomatics
- [ ] Radiology
- [ ] Nuclear Medicine
- [ ] Ophthalmology
- [ ] Otorhinolaryngology
- [ ] Dermatology
- [ ] Anatomy
- [ ] Occupational Medicine
- [ ] Environmental Medicine
- [ ] Human Genetics
- [ ] Microbiology and Virology
- [ ] Pathology
- [ ] Pharmacology

Survey generated with
LamaPoll

- [ ] Phoniatrics
- [ ] Forensic Medicine
- [ ] Other

Survey generated with
LamaPoll

Clinical Traineeship Preparatory Course - PRE

ECG Segment

	Yes	No
As part of my course of study, I already have practical experience in reading ECGs, e.g., in seminars.	◯	◯
I already have practical experience reading ECGs in clinical settings, e.g., as part of clinical traineeships).	◯	◯

Survey generated with
LamaPoll

Please rate the following statements.

	Disagree completely					Agree completely
The ECG is an important diagnostic tool in medical practice.	☐	☐	☐	☐	☐	☐
Medical students should learn early on how an ECG is structured and independently interpret the results.	☐	☐	☐	☐	☐	☐
Every physician (regardless of specialty) should be able to interpret an ECG in a structured manner.	☐	☐	☐	☐	☐	☐
As part of my studies I wish to have practical exercises in interpreting ECGs.	☐	☐	☐	☐	☐	☐
Interpreting ECGs in a structured manner is a task for medical students within the scope of a clinical practicums, such as the traineeship (evaluation also by supervising physician).	☐	☐	☐	☐	☐	☐
I can explain the basic differences between Einthoven, Goldberger and Wilson leads.	☐	☐	☐	☐	☐	☐
I can independently do a 12-channel ECG (electrode placement and leads).	☐	☐	☐	☐	☐	☐
I can recognize a physiological ECG and identify the characteristics of a physiological ECG.	☐	☐	☐	☐	☐	☐
I can determine the axis based on an ECG and understand the separate steps (understand the physiological principles instead of memorizing the steps).	☐	☐	☐	☐	☐	☐
I know how to proceed with a structured ECG finding.	☐	☐	☐	☐	☐	☐
I can recognize and identify the basic pathological signs of atrial fibrillation on an ECG.	☐	☐	☐	☐	☐	☐
I can recognize and identify the basic pathological signs of ventricular and supraventricular extrasystoles on an ECG.	☐	☐	☐	☐	☐	☐
I can recognize and identify the basic pathological signs of an AV block on an ECG.	☐	☐	☐	☐	☐	☐

Survey generated with
LamaPoll

I can recognize the basic pathological signs of a myocardial infarct on an ECG and determine the location of the infarct.

☐ ☐ ☐ ☐ ☐ ☐

Learning a standardized interpretation procedure helps me to structure the results of ECGs.

☐ ☐ ☐ ☐ ☐ ☐

How often have you performed a (structured) interpretation of ECG results?

- ◯ 0
- ◯ 1-3
- ◯ 4-5
- ◯ 6-10
- ◯ 11-20
- ◯ > 20

Survey generated with LamaPoll

Clinical Traineeship Preparatory Course - PRE

Experience with Simulated Patients

	Yes	No
Have you already had training in anamnesis with simulated patients.	○	○
Have you already taken another course that used simulated patients?	○	○

Survey generated with LamaPoll

Subjective Confidence

Please rate how confident you currently feel when performing the following tasks:

	Disagree completely					Agree completely
How confident do you feel in taking a structured case history?	☐	☐	☐	☐	☐	☐
How confident do you feel in using the ISOBAR checklist?	☐	☐	☐	☐	☐	☐
How confident do you feel in communicating appropriately with a patient?	☐	☐	☐	☐	☐	☐
How confident do you feel in making a precise and accurate medical handover?	☐	☐	☐	☐	☐	☐
How confident do you feel in communicating appropriately with members of the other healthcare professions?	☐	☐	☐	☐	☐	☐

Survey generated with
LamaPoll
