# Supplementary material for: Telemedicine in medical education: An example of a digital preparatory course for the clinical traineeship – a pre-post comparison
Source: GMS J Med Educ. 2022 Sep 15;39(4):Doc46. doi: 10.3205/zma001567 (PMC9585416; doi:10.3205/zma001567)
Supplement: Post-evaluation [file JME-39-46-s-007.pdf]

Attachment 7: Post-evaluation

Attachment 7 to Vogt L, Schmidt M, Follmann A, Lenes A, Klasen M, Sopka S. *Telemedicine in medical education: An example of a digital preparatory course for the clinical traineeship – a pre-post comparison*. GMS J Med Educ. 2022;39(4):Doc46. DOI: 10.3205/zma001567

Clinical Traineeship Preparatory Course - POST

Dear students,

Thank you for taking the time to fill out this questionnaire.
The aim is to evaluate our Clinical Traineeship Preparatory Course at Aixtra and to adapt the teaching to the needs of the students.
This survey will take about 10 minutes.
Participation is voluntary and anonymous. No one will be able to match you with your answers.
If you have any questions, we will be happy to answer them. Thank you for your participation!

AIXTRA Team

Personal Code

Please enter your **personal code** according to the following instructions:

First letter of your mother's first name
First letter of your father's first name
Your mother's day of birth
Your father's day of birth

Example:
Mother's first name: Anna => A
Father's first name: Max => M
Mother's day of birth: March 5th => 05
Father's day of birth: November 12th => 12
Example code: AM0512

Personal code

Survey generated with
LamaPoll

Course date

Please select the date of your course:

Date ☐

General Questions about the Clinical Traineeship Preparatory Course

Please rate the following statements.

	Disagree completely					Agree completely
My expectations for this AIXTRA preparatory course were met.	☐	☐	☐	☐	☐	☐
The instructors/tutors encouraged me to actively participate.	☐	☐	☐	☐	☐	☐
The course learning objectives were clearly defined.	☐	☐	☐	☐	☐	☐
The presentation of the learning material was understandable.	☐	☐	☐	☐	☐	☐
A uniform teaching strategy was visible in the teaching of practical skills.	☐	☐	☐	☐	☐	☐
The practice time was sufficient for me.	☐	☐	☐	☐	☐	☐
I don't need the practiced skills for the clinical traineeship.	☐	☐	☐	☐	☐	☐
The atmosphere was predominantly constructive.	☐	☐	☐	☐	☐	☐

Survey generated with
LamaPoll

ECG Segment

Please rate the following statements on the course segment dealing with ECGs.

	Disagree completely					Agree completely
I can explain the basic differences between Einthoven, Goldberger and Wilson leads.	☐	☐	☐	☐	☐	☐
I can independently do a 12-channel ECG (electrode placement and leads).	☐	☐	☐	☐	☐	☐
I can recognize a physiological ECG and identify the characteristics of a physiological ECG.	☐	☐	☐	☐	☐	☐
I can determine the axis based on an ECG and understand the separate steps (understand the physiological principles instead of memorizing the steps).	☐	☐	☐	☐	☐	☐
I know how to proceed with a structured ECG finding.	☐	☐	☐	☐	☐	☐
I can recognize and identify the basic pathological signs of atrial fibrillation on an ECG.	☐	☐	☐	☐	☐	☐
I can recognize and identify the basic pathological signs of ventricular and supraventricular extrasystoles on an ECG.	☐	☐	☐	☐	☐	☐
I can recognize and identify the basic pathological signs of an AV block on an ECG.	☐	☐	☐	☐	☐	☐
I can recognize the basic pathological signs of a myocardial infarct on an ECG and determine the location of the infarct.	☐	☐	☐	☐	☐	☐
Learning a standardized interpretation procedure helps me to structure the results of ECGs.	☐	☐	☐	☐	☐	☐

Evaluation of the ECG Segment

Please rate the following statements on the course segment dealing with ECGs.

	Disagree completely					Agree completely
I have improved my understanding of ECG basic as a result of the ECG course.	☐	☐	☐	☐	☐	☐
I feel more confident in interpreting an ECG result as a result of the ECG course.	☐	☐	☐	☐	☐	☐
My expectations for the ECG course were met.	☐	☐	☐	☐	☐	☐
The ECG course was clearly structured in its organization.	☐	☐	☐	☐	☐	☐
The theoretical part of the ECG course was appropriate.	☐	☐	☐	☐	☐	☐
The theoretical principles were taught in an understandable way.	☐	☐	☐	☐	☐	☐
The time for independent practice during the course was sufficient.	☐	☐	☐	☐	☐	☐
The scope of the learning materials was appropriate.	☐	☐	☐	☐	☐	☐
The important points in the course materials were made clear.	☐	☐	☐	☐	☐	☐
The level of the ECG course was appropriate.	☐	☐	☐	☐	☐	☐
The test questions were appropriate.	☐	☐	☐	☐	☐	☐

Simulated Patient Segment

Today I performed a practical anamnesis exercise with a simulated patient.

○ Yes ○ No

Evaluation of the Segment with Simulated Patients

Please rate the following statements on the course segment with simulated patients.

	Disagree completely					Agree completely
I learned how to build a solid doctor-patient relationship.	☐	☐	☐	☐	☐	☐
I learned how to take a structured case history.	☐	☐	☐	☐	☐	☐
I learned how to explain the course of further action to a patient.	☐	☐	☐	☐	☐	☐
The anamnesis training was important preparation for my upcoming clinical traineeship.	☐	☐	☐	☐	☐	☐
The feedback from the simulated patient was helpful for me.	☐	☐	☐	☐	☐	☐
The feedback of my fellow students was helpful for me.	☐	☐	☐	☐	☐	☐
The feedback from the instructors was helpful for me.	☐	☐	☐	☐	☐	☐
The anamnesis training was important preparation for my upcoming clinical traineeship.	☐	☐	☐	☐	☐	☐
The instructor clearly structured the anamnesis training session.	☐	☐	☐	☐	☐	☐
The instructor was open to answering questions.	☐	☐	☐	☐	☐	☐
The instructor gave satisfactory answers to the questions that were asked.	☐	☐	☐	☐	☐	☐

Subjective Confidence

Please rate how confident you currently feel when performing the following tasks:

	Disagree completely					Agree completely
How confident do you feel in taking a structured case history?	☐	☐	☐	☐	☐	☐
How confident do you feel in using the ISOBAR checklist?	☐	☐	☐	☐	☐	☐
How confident do you feel in communicating appropriately with a patient?	☐	☐	☐	☐	☐	☐
How confident do you feel in making a precise and accurate medical handover?	☐	☐	☐	☐	☐	☐
How confident do you feel in communicating appropriately with members of the other healthcare professions?	☐	☐	☐	☐	☐	☐

Survey generated with
LamaPoll

Comments and Suggestions

I really liked the following about this course:

I did not like the following about this course:

I have these suggestions for improving the course:

Other comments:

I give the Clinical Traineeship Preparatory Course the following overall score (conventional German academic grading scale):

Please assign one grade for the course (1 = "excellent", 6 = "deficient / very poor"):

- ◯ 1
- ◯ 2
- ◯ 3
- ◯ 4
- ◯ 5
- ◯ 6

Survey generated with
LamaPoll
